# *N*,*N*′-Diphenyl­but-2-enediamide

**DOI:** 10.1107/S1600536810051330

**Published:** 2010-12-11

**Authors:** B. Thimme Gowda, Sabine Foro, K. Shakuntala, Hartmut Fuess

**Affiliations:** a Department of Chemistry, Mangalore University, Mangalagangotri 574 199, Mangalore, India; b Institute of Materials Science, Darmstadt University of Technology, Petersenstrasse 23, D-64287 Darmstadt, Germany

## Abstract

In the title compound, C_16_H_14_N_2_O_2_, the conformations of the N—H and C=O bonds in the C—NH—CO—CH =CH—CO—NH—C segment are *anti* to each other. The two C=O bonds are also *anti* to each other. The two phenyl rings make an inter­planar angle of 41.2 (1)°. An intra­molecular N—H⋯O hydrogen bond occurs. In the crystal, inter­molecular N—H⋯O hydrogen bonding links the mol­ecules into infinite chains along the *a* axis.

## Related literature

For related structures, see: Gowda, Foro *et al.* (2010 ▶); Gowda, Tokarčík *et al.* (2010 ▶).
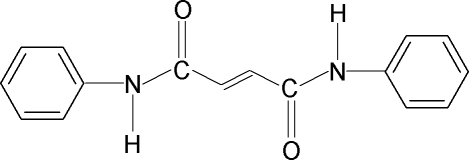


## Experimental

### 

#### Crystal data

C_16_H_14_N_2_O_2_
*M* *_r_* = 266.29
Orthorhombic, 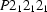

*a* = 6.604 (1) Å
*b* = 13.358 (2) Å
*c* = 15.474 (2) Å
*V* = 1365.1 (3) Å^3^
*Z* = 4
Cu *K*α radiation
μ = 0.70 mm^−1^
*T* = 299 K
0.35 × 0.30 × 0.25 mm

#### Data collection

Enraf–Nonius CAD-4 diffractometer
3656 measured reflections
1422 independent reflections
1339 reflections with *I* > 2σ(*I*)
*R*_int_ = 0.117
3 standard reflections every 120 min  intensity decay: 0.5%

#### Refinement

*R*[*F* ^2^ > 2σ(*F* ^2^)] = 0.043
*wR*(*F* ^2^) = 0.100
*S* = 1.06
1422 reflections
188 parameters
2 restraints
H atoms treated by a mixture of independent and constrained refinement
Δρ_max_ = 0.11 e Å^−3^
Δρ_min_ = −0.13 e Å^−3^

### 

Data collection: *CAD-4-PC* (Enraf–Nonius, 1996 ▶); cell refinement: *CAD-4-PC*; data reduction: *REDU4* (Stoe & Cie, 1987 ▶); program(s) used to solve structure: *SHELXS97* (Sheldrick, 2008 ▶); program(s) used to refine structure: *SHELXL97* (Sheldrick, 2008 ▶); molecular graphics: *PLATON* (Spek, 2009 ▶); software used to prepare material for publication: *SHELXL97*.

## Supplementary Material

Crystal structure: contains datablocks I, global. DOI: 10.1107/S1600536810051330/ng5082sup1.cif

Structure factors: contains datablocks I. DOI: 10.1107/S1600536810051330/ng5082Isup2.hkl

Additional supplementary materials:  crystallographic information; 3D view; checkCIF report

## Figures and Tables

**Table 1:** Hydrogen-bond geometry (Å, °)

*D*—H⋯*A*	*D*—H	H⋯*A*	*D*⋯*A*	*D*—H⋯*A*
N1—H1*N*⋯O2^i^	0.86 (2)	2.04 (2)	2.884 (3)	167 (2)
N2—H2*N*⋯O1	0.91 (2)	1.77 (2)	2.671 (3)	167 (3)

Symmetry code: (i) 
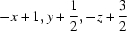
.

**Fig. 1.**
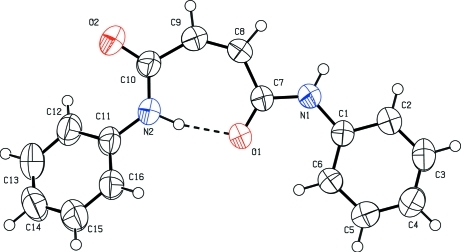
Molecular structure of (I), showing the atom labelling scheme and displacement ellipsoids are drawn at the 50% probability level.

**Fig. 2.**
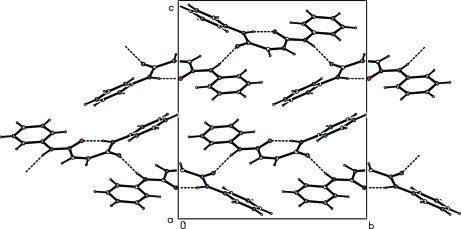
Molecular packing of (I) with hydrogen bonding shown as dashed lines.

## supplementary crystallographic information

### Comment

The amide moiety is an important constituent of many biologically significant compounds. As a part of studying the effect of substitutions on the structures of this class of compounds (Gowda, Foro *et al.*, 2010; Gowda, Tokarčík *et al.*, 2010), the crystal structure of *N*,*N*-bis(phenyl)-maleamide has been determined (I) (Fig. 1).

In the structure, the conformations of N—H and C=O bonds in both the amide groups of the C—NH—CO—CH ═CH—CO—NH—C segment are *anti* to each other. The two C=O bonds are also *anti* to each other, while one of them is *syn* to the adjacent C—H bond and the other is *anti* to its adjacent C—H bond. Further, C1—N1—C7—C8 and C11—N2—C10—C9 segments are nearly linear and so also the C1—N1—C7—O1 and C11—N2—C10—O2 segments. The torsion angles of C2—C1—N1—C7 and C6—C1—N1—C7 are 174.4 (3)° and -4.9 (4)°, respectively, while those of C12—C11—N2—C10 and C16—C11—N2—C10 are 40.4 (4)° and -143.9 (3)°.

The two phenyl rings in (I) make an interplanar angle of 41.2 (1)°. while the two benzene rings (C1 to C6 and C11 to C16) make the dihedral angles of 8.0 (1)° and 38.3 (1)°, respectively, with the central amide group (N1—C7—C8—C9—C10—N2).

The crystal structure (Fig. 2) exhibits both the intramolecular and intermolecular N–H···O hydrogen bonds (Table 1). The latter link the molecules into chains.

### Experimental

A mixture of maleic acid (0.2 mol) and phosphorous oxy chloride (0.3 mol) were refluxed for 3 hrs on a water bath at 95° C. The aniline was added dropwise with stirring and continuing heating for about 30 min. It was later kept aside for 12 hrs for completion of the reaction. The reaction mixture was then added to ice. The precipitated product was washed with water, dilute HCl, dilute NaOH and again with water. The product was filtered, dried and recrystallized from DMF.

Prism like orange single crystals of the title compound used in X-ray diffraction studies were obtained by a slow evaporation of its DMF solution at room temperature.

### Refinement

The H atoms of the NH groups were located in a difference map and later restrained to the distance N—H = 0.86 (2) Å. The other H atoms were positioned with idealized geometry using a riding model with C—H = 0.93 Å A l l H atoms were refined with isotropic displacement parameters (set to 1.2 times of the *U*_eq_ of the parent atom).

In the absence of significant anomalous dispersion effects, Friedel pairs were merged and the Δf"term set to zero.

### Crystal data

**Table:**

C_16_H_14_N_2_O_2_	*F*(000) = 560
*M**_r_* = 266.29	*D*_x_ = 1.296 Mg m^−^^3^
Orthorhombic, *P*2_1_2_1_2_1_	Cu *K*α radiation, λ = 1.54180 Å
Hall symbol: P 2ac 2ab	Cell parameters from 25 reflections
*a* = 6.604 (1) Å	θ = 8.0–20.1°
*b* = 13.358 (2) Å	µ = 0.70 mm^−^^1^
*c* = 15.474 (2) Å	*T* = 299 K
*V* = 1365.1 (3) Å^3^	Prism, orange
*Z* = 4	0.35 × 0.30 × 0.25 mm

### Data collection

**Table:**

Enraf–Nonius CAD-4 diffractometer	*R*_int_ = 0.117
Radiation source: fine-focus sealed tube	θ_max_ = 66.9°, θ_min_ = 4.4°
graphite	*h* = 0→7
ω scans	*k* = −15→15
3656 measured reflections	*l* = −18→5
1422 independent reflections	3 standard reflections every 120 min
1339 reflections with *I* > 2σ(*I*)	intensity decay: 0.5%

### Refinement

**Table:**

Refinement on *F*^2^	Secondary atom site location: difference Fourier map
Least-squares matrix: full	Hydrogen site location: inferred from neighbouring sites
*R*[*F*^2^ > 2σ(*F*^2^)] = 0.043	H atoms treated by a mixture of independent and constrained refinement
*wR*(*F*^2^) = 0.100	*w* = 1/[σ^2^(*F*_o_^2^) + (0.0279*P*)^2^ + 0.0657*P*] where *P* = (*F*_o_^2^ + 2*F*_c_^2^)/3
*S* = 1.06	(Δ/σ)_max_ = 0.025
1422 reflections	Δρ_max_ = 0.11 e Å^−^^3^
188 parameters	Δρ_min_ = −0.13 e Å^−^^3^
2 restraints	Extinction correction: *SHELXL97* (Sheldrick, 2008), Fc^*^=kFc[1+0.001xFc^2^λ^3^/sin(2θ)]^-1/4^
Primary atom site location: structure-invariant direct methods	Extinction coefficient: 0.0140 (9)

### Special details

**Table:**

Geometry. All e.s.d.'s (except the e.s.d. in the dihedral angle between two l.s. planes) are estimated using the full covariance matrix. The cell e.s.d.'s are taken into account individually in the estimation of e.s.d.'s in distances, angles and torsion angles; correlations between e.s.d.'s in cell parameters are only used when they are defined by crystal symmetry. An approximate (isotropic) treatment of cell e.s.d.'s is used for estimating e.s.d.'s involving l.s. planes.
Refinement. Refinement of *F*^2^ against ALL reflections. The weighted *R*-factor *wR* and goodness of fit *S* are based on *F*^2^, conventional *R*-factors *R* are based on *F*, with *F* set to zero for negative *F*^2^. The threshold expression of *F*^2^ > σ(*F*^2^) is used only for calculating *R*-factors(gt) *etc*. and is not relevant to the choice of reflections for refinement. *R*-factors based on *F*^2^ are statistically about twice as large as those based on *F*, and *R*- factors based on ALL data will be even larger.

### Fractional atomic coordinates and isotropic or equivalent isotropic displacement parameters (Å^2^)

**Table:**

	*x*	*y*	*z*	*U*_iso_*/*U*_eq_	
C1	−0.0250 (3)	0.71987 (19)	0.85351 (17)	0.0547 (6)	
C2	−0.0384 (3)	0.8224 (2)	0.8469 (2)	0.0678 (7)	
H2	0.0653	0.8579	0.8202	0.081*	
C3	−0.2036 (4)	0.8730 (2)	0.8794 (2)	0.0761 (8)	
H3	−0.2105	0.9424	0.8747	0.091*	
C4	−0.3580 (4)	0.8212 (3)	0.9186 (2)	0.0760 (8)	
H4	−0.4702	0.8550	0.9402	0.091*	
C5	−0.3449 (3)	0.7192 (3)	0.9256 (2)	0.0750 (9)	
H5	−0.4490	0.6841	0.9525	0.090*	
C6	−0.1793 (3)	0.6671 (2)	0.89343 (17)	0.0630 (7)	
H6	−0.1722	0.5978	0.8986	0.076*	
C7	0.2087 (3)	0.57754 (19)	0.82283 (19)	0.0610 (7)	
C8	0.4088 (3)	0.5604 (2)	0.78135 (18)	0.0653 (7)	
H8	0.4646	0.6163	0.7545	0.078*	
C9	0.5210 (3)	0.4774 (2)	0.7765 (2)	0.0683 (8)	
H9	0.6417	0.4879	0.7469	0.082*	
C10	0.4997 (3)	0.3727 (2)	0.80698 (18)	0.0597 (6)	
C11	0.2874 (3)	0.2512 (2)	0.88312 (16)	0.0581 (6)	
C12	0.4328 (4)	0.1895 (2)	0.92070 (19)	0.0731 (8)	
H12	0.5675	0.2098	0.9228	0.088*	
C13	0.3764 (4)	0.0982 (3)	0.9549 (2)	0.0839 (9)	
H13	0.4740	0.0570	0.9796	0.101*	
C14	0.1789 (4)	0.0676 (3)	0.9529 (2)	0.0882 (9)	
H14	0.1423	0.0059	0.9758	0.106*	
C15	0.0352 (4)	0.1288 (2)	0.9168 (2)	0.0856 (9)	
H15	−0.0994	0.1083	0.9158	0.103*	
C16	0.0868 (3)	0.2196 (2)	0.8821 (2)	0.0687 (7)	
H16	−0.0124	0.2603	0.8578	0.082*	
N1	0.1514 (2)	0.67393 (16)	0.81901 (16)	0.0596 (6)	
H1N	0.227 (3)	0.7171 (19)	0.7929 (15)	0.071*	
N2	0.3325 (2)	0.34685 (16)	0.85126 (16)	0.0617 (6)	
H2N	0.241 (3)	0.3975 (19)	0.8579 (19)	0.074*	
O1	0.1061 (2)	0.51235 (14)	0.85749 (17)	0.0956 (8)	
O2	0.6377 (2)	0.31390 (16)	0.79069 (14)	0.0769 (6)	

### Atomic displacement parameters (Å^2^)

**Table:**

	*U*^11^	*U*^22^	*U*^33^	*U*^12^	*U*^13^	*U*^23^
C1	0.0536 (9)	0.0455 (12)	0.0648 (13)	0.0038 (9)	0.0018 (10)	0.0006 (15)
C2	0.0679 (10)	0.0500 (13)	0.0856 (18)	0.0021 (11)	0.0090 (13)	0.0060 (19)
C3	0.0837 (14)	0.0501 (14)	0.0944 (18)	0.0131 (12)	−0.0005 (16)	0.003 (2)
C4	0.0719 (12)	0.0706 (18)	0.0856 (19)	0.0169 (14)	0.0090 (14)	0.003 (2)
C5	0.0609 (11)	0.0733 (19)	0.091 (2)	0.0053 (11)	0.0134 (13)	0.009 (2)
C6	0.0547 (9)	0.0530 (14)	0.0812 (16)	0.0001 (10)	0.0045 (11)	0.0110 (18)
C7	0.0570 (9)	0.0424 (12)	0.0836 (17)	0.0014 (9)	0.0108 (12)	0.0025 (17)
C8	0.0576 (10)	0.0479 (12)	0.0903 (17)	−0.0045 (10)	0.0169 (12)	0.0066 (18)
C9	0.0561 (9)	0.0573 (15)	0.091 (2)	0.0007 (10)	0.0161 (13)	0.0009 (19)
C10	0.0553 (10)	0.0499 (13)	0.0740 (15)	0.0027 (9)	0.0055 (11)	−0.0068 (17)
C11	0.0680 (10)	0.0448 (12)	0.0616 (13)	0.0093 (10)	0.0031 (11)	−0.0048 (16)
C12	0.0715 (12)	0.0619 (15)	0.0860 (18)	0.0168 (13)	−0.0039 (13)	0.004 (2)
C13	0.0977 (17)	0.0612 (17)	0.093 (2)	0.0209 (16)	−0.0119 (17)	0.009 (2)
C14	0.1098 (18)	0.0530 (15)	0.102 (2)	−0.0005 (16)	0.0011 (18)	0.017 (2)
C15	0.0820 (14)	0.0579 (16)	0.117 (3)	−0.0023 (13)	−0.0005 (16)	0.003 (2)
C16	0.0657 (12)	0.0547 (14)	0.0856 (18)	0.0070 (11)	−0.0023 (13)	0.0048 (19)
N1	0.0511 (8)	0.0435 (10)	0.0841 (14)	−0.0005 (8)	0.0082 (9)	0.0044 (14)
N2	0.0568 (8)	0.0468 (11)	0.0816 (14)	0.0093 (8)	0.0079 (10)	0.0028 (14)
O1	0.0772 (9)	0.0461 (9)	0.164 (2)	0.0100 (8)	0.0493 (12)	0.0271 (16)
O2	0.0675 (8)	0.0636 (12)	0.0995 (13)	0.0182 (9)	0.0157 (9)	−0.0071 (14)

### Geometric parameters (Å, °)

**Table:**

C1—C2	1.376 (4)	C9—H9	0.9300
C1—C6	1.385 (3)	C10—O2	1.230 (3)
C1—N1	1.421 (3)	C10—N2	1.345 (3)
C2—C3	1.379 (4)	C11—C16	1.390 (3)
C2—H2	0.9300	C11—C12	1.392 (3)
C3—C4	1.374 (4)	C11—N2	1.402 (3)
C3—H3	0.9300	C12—C13	1.381 (5)
C4—C5	1.370 (5)	C12—H12	0.9300
C4—H4	0.9300	C13—C14	1.367 (4)
C5—C6	1.388 (3)	C13—H13	0.9300
C5—H5	0.9300	C14—C15	1.372 (4)
C6—H6	0.9300	C14—H14	0.9300
C7—O1	1.227 (3)	C15—C16	1.370 (4)
C7—N1	1.344 (3)	C15—H15	0.9300
C7—C8	1.487 (3)	C16—H16	0.9300
C8—C9	1.335 (4)	N1—H1N	0.862 (18)
C8—H8	0.9300	N2—H2N	0.912 (18)
C9—C10	1.482 (4)		
			
C2—C1—C6	119.5 (2)	O2—C10—N2	123.3 (3)
C2—C1—N1	117.0 (2)	O2—C10—C9	117.9 (2)
C6—C1—N1	123.4 (2)	N2—C10—C9	118.8 (2)
C1—C2—C3	120.8 (3)	C16—C11—C12	118.9 (3)
C1—C2—H2	119.6	C16—C11—N2	118.3 (2)
C3—C2—H2	119.6	C12—C11—N2	122.7 (2)
C4—C3—C2	120.1 (3)	C13—C12—C11	119.8 (2)
C4—C3—H3	120.0	C13—C12—H12	120.1
C2—C3—H3	120.0	C11—C12—H12	120.1
C5—C4—C3	119.3 (3)	C14—C13—C12	120.8 (3)
C5—C4—H4	120.3	C14—C13—H13	119.6
C3—C4—H4	120.3	C12—C13—H13	119.6
C4—C5—C6	121.3 (3)	C13—C14—C15	119.4 (3)
C4—C5—H5	119.3	C13—C14—H14	120.3
C6—C5—H5	119.3	C15—C14—H14	120.3
C1—C6—C5	119.0 (3)	C16—C15—C14	121.0 (3)
C1—C6—H6	120.5	C16—C15—H15	119.5
C5—C6—H6	120.5	C14—C15—H15	119.5
O1—C7—N1	122.9 (2)	C15—C16—C11	120.1 (3)
O1—C7—C8	124.8 (2)	C15—C16—H16	120.0
N1—C7—C8	112.3 (2)	C11—C16—H16	120.0
C9—C8—C7	130.1 (3)	C7—N1—C1	129.0 (2)
C9—C8—H8	114.9	C7—N1—H1N	119.9 (18)
C7—C8—H8	114.9	C1—N1—H1N	111.1 (18)
C8—C9—C10	135.5 (2)	C10—N2—C11	126.0 (2)
C8—C9—H9	112.3	C10—N2—H2N	114.2 (19)
C10—C9—H9	112.3	C11—N2—H2N	119.8 (18)
			
C6—C1—C2—C3	−0.1 (5)	C11—C12—C13—C14	−0.5 (5)
N1—C1—C2—C3	−179.4 (2)	C12—C13—C14—C15	−0.3 (5)
C1—C2—C3—C4	−0.3 (5)	C13—C14—C15—C16	0.5 (5)
C2—C3—C4—C5	0.5 (5)	C14—C15—C16—C11	0.0 (5)
C3—C4—C5—C6	−0.4 (6)	C12—C11—C16—C15	−0.7 (4)
C2—C1—C6—C5	0.2 (4)	N2—C11—C16—C15	−176.6 (3)
N1—C1—C6—C5	179.5 (2)	O1—C7—N1—C1	1.8 (5)
C4—C5—C6—C1	0.0 (5)	C8—C7—N1—C1	−177.7 (2)
O1—C7—C8—C9	−3.9 (5)	C2—C1—N1—C7	174.4 (3)
N1—C7—C8—C9	175.6 (3)	C6—C1—N1—C7	−4.9 (4)
C7—C8—C9—C10	0.5 (6)	O2—C10—N2—C11	−1.7 (4)
C8—C9—C10—O2	−179.8 (3)	C9—C10—N2—C11	178.8 (3)
C8—C9—C10—N2	−0.3 (6)	C16—C11—N2—C10	−143.9 (3)
C16—C11—C12—C13	1.0 (4)	C12—C11—N2—C10	40.4 (4)
N2—C11—C12—C13	176.6 (3)		

### Hydrogen-bond geometry (Å, °)

**Table:**

*D*—H···*A*	*D*—H	H···*A*	*D*···*A*	*D*—H···*A*
N1—H1N···O2^i^	0.86 (2)	2.04 (2)	2.884 (3)	167 (2)
N2—H2N···O1	0.91 (2)	1.77 (2)	2.671 (3)	167 (3)

Symmetry codes: (i) −*x*+1, *y*+1/2, −*z*+3/2.
